# Compressions of magnetorheological fluids under instantaneous magnetic field and constant area

**DOI:** 10.1038/s41598-021-88407-0

**Published:** 2021-04-26

**Authors:** Hongyun Wang, Cheng Bi, Yongju Zhang, Li Zhang, Fenfen Zhou

**Affiliations:** College of Aeronautics, Taizhou University, Taizhou, 318000 Zhejiang China

**Keywords:** Engineering, Materials science, Nanoscience and technology

## Abstract

Compressions of magnetorheological (MR) fluids have been carried out under instantaneous magnetic fields. The yield strength of the MR fluid in compressive mode has been derived by assuming that it was a transformed shear flow in Bi-visous model. The compressive stresses have experimentally studied under different magnetic fields, different initial gap distances and different compressive velocities. The nominal yield shear stresses of the compressed MR fluid under different influential factors have been calculated. The compressive stress increased in a power law as the applied magnetic field increased, while it decreased as the initial gap distance and the compressive velocity increased. With the increase of magnetic field, the difference between the nominal yield shear stress curves increased, and the exponents of the power law increased with the increase of the magnetic field strengths. A larger initial gap distance and a lower compressive velocity resulted in a higher nominal yield shear stress under the same instantaneous magnetic field. The achieved results of the nominal yield shear stress with magnetic field seemed to deviate from the prediction of dipole model, and the chain structure aggregation effect, the sealing effect and the friction effect by compression should be considered.

## Introduction

Magnetorheological (MR) fluids are a class of smart material and have been greatly investigated by industrial and academic communities for its peculiar performance. So they have many industrial applications such as clutches, brakes, dampers and actuators. According to the working mode of MR fluids, applications of the device can be classified as the valve mode, the shear mode, the squeeze mode or a combination of these modes. Researchers have thoroughly studied the dynamic and steady performance of MR fluids under shear mode^1–3^. The shear yield stress of MR fluids is the most important parameter for its applications under shear mode. A higher shear yield stress of MR fluids means a higher mechanical performance of MR device. However, the real applications of MR device are restricted due to the capacity of shear yield stress of MR fluids. Thus, great efforts have been made to develop a new MR fluids by many researchers^4^. Except for inventing a new MR fluids with high performance, Tang et al. first found that MR fluids squeezed may provide a ten times higher yield shear stress than sheared under the same magnetic field, which was usually explained by the squeeze-strengthen effect of MR fluids^5^. It is another method of improving the shear yield stress of MR fluids. Subsequently, the squeeze-strengthen effect was further demonstrated own to the formation of thick columns with strong and robust ends under compression by Zhang et al.^6^. See et al. have investigated the pre-compression after applying an magnetic field but before shearing, showing that compression did not improve the yield shear stress of MR fluids^7,8^, which is contrary to what Zhang et al. believed. Kulkarni et al*.* have experimentally studied the behavior of MR fluids in squeeze, and have found that the introduction of squeeze in the shear mode does not always increase the yield stress of MR fluids^9^. Mazlan et al*.* have reported that the compressive stress is dependent on the magnetic field and the gap size, but the compressive velocity has no significant effect on the stress–strain curves^10,11^. Ruiz-López et al. have proposed a micromechanical model and have presented an extensive experimental investigation of normal force versus 1 − *ε* (*ε* is the compressive strain) in unidirectional slow-compression, no-slip, constant-volume squeeze mode under different magnetic field strengths, viscosities and particle concentrations^12,13^. Guo et al. have studied that the normal force versus the gap under the constant volume and the uniform magnetic field with a self-developed device^14^. They have found that a smaller initial gap distance can obtain larger normal force at the same strain, which is contrary to the results of Mazlan et al*.*^10^. The relation between the normal force and the gap/1 − *ε* with exponent in the range (− 3, − 2) has be obtained^12–14^. We have experimentally studied compression, elongation and shearing behaviors of MR fluid, showing and the compressive stress is much higher than the shear yield stress^15^. Li et al*.* have established the MRF squeeze flow theory model, and found loading speed, magnetic field change gradient, and magnetorheological fluid dynamic yield strength are the key factors affecting the squeeze force^16^.

For all the reported squeezing mode, including self-assembled devices^5,6,9,10,14,15^ or commercial setups^7,8,12,13^, the magnetic field strength was usually dealt with as a constant value during the compression. However, the magnetic field density is not constant under a constant current during the compression, and it increases with the decrease of the gap during the compression because the magnetic resistance associated to the gap is modified. Any change in the structure parameter (the magnetic field, volume fraction, and structural strength i.e.) will affect the compressive properties. The magnetic field strength would change the properties of MR fluid. Therefore, the compressive properties of MR fluids are inevitably affected by the instantaneous magnetic field. In this paper, the compressive behaviors of MR fluids under different magnetic fields, different compressive velocities, different initial gap distances, and at the instantaneous magnetic field were investigated. Deviations from the traditional description have been found and discussed.

## Theoretical analysis

In order to describe the rheological properties of MR fluid in the pre-yield zone, Bi-visous model is adopted^17–20^. The shear stress is given by

1 $$ \left\{ \begin{gathered} \tau = \eta \frac{du}{{dz}}\quad \quad \quad \quad \quad \left| \tau \right| < \tau_{d} \hfill \\ \tau = \tau_{0} + \eta_{H} \frac{du}{{dz}}\quad \,\,\quad \left| \tau \right| \ge \tau_{d} \hfill \\ \end{gathered} \right. $$

where *τ*_*d*_ is the dynamic yield shear stress that is a function of magnetic flux density *B* as *τ*_*d*_ = *αB*^*n*^ (*α*,*n* are the constants related to the properties of MR fluid), *τ*_*0*_ is the yield shear stress, *η* and *η*_*H*_ are the pre-yield and post-yield viscosity in the Bi-visous model, respectively. When |*τ*| <  *τ*_*d*_ under the applied magnetic field, chain-like structures in MR fluids are formed and flowed very slowly with very high viscosity *η*. The coefficient of viscosity *k* is a very important parameter in the Bi-visous model and it is the ratio of *η*_*H*_ and *η*. Normally, *k* is the value of 10^–5^–10^–2^ and $\tau_{d} = \tau_{0} \left( {1 - k} \right)$.

**Figure 1:**
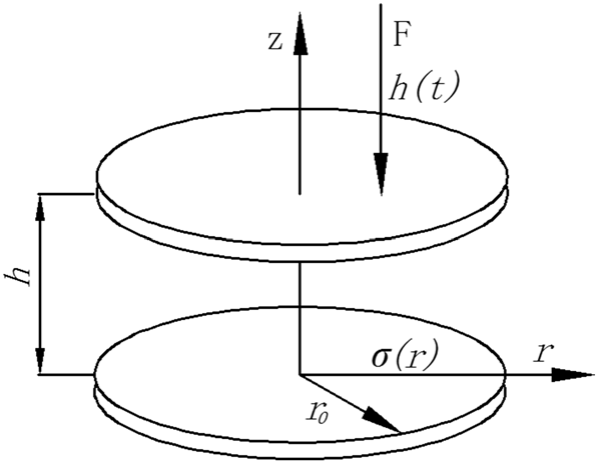
The sketch of the compression of MR fluids between two parallel plates.

The squeeze flow of MR fluids between two parallel plates with a radius *r* and a gap distance *h* is showed in Fig. 1. The upper plate moves slowly at the speed of *dh/dt* along the *z* direction toward the static bottom plate. Because of a low compressive velocity, the mass force of the fluid can be neglected. According to lubrication theory, the compressive stress can be predicted by^18,19^

2 $$ \frac{d\sigma }{{dr}} = \frac{d\tau }{{dz}} $$

where *dσ/dr* is the pressure gradient along the radius *r, τ* is shear stress of MR fluids at the position. The radial pressure distribution can be represented as

3 $$ \sigma \left( r \right) = \int_{{r_{0} }}^{r} {d\sigma \left( r \right)} $$

The compressive force *F* acting on the plates can be obtained by integration on the surface

4 $$ F = \int_{0}^{{r_{0} }} {\sigma \left( r \right)} \cdot 2\pi rdr $$

According to the description of Williams et al*.*^18^, the compressive force *F* can be represented as

5 $$ F = \frac{{2\pi \tau_{y} R^{3} }}{{hS^{3} }}\left[ {\frac{{k^{3} }}{108} + \int_{{{\raise0.7ex\hbox{$k$} \!\mathord{\left/ {\vphantom {k 3}}\right.\kern-\nulldelimiterspace} \!\lower0.7ex\hbox{$3$}}}}^{S} {S^{2} \left( { - \frac{h}{{2\tau_{y} }}\frac{d\sigma }{{dr}}} \right)} dS} \right] $$

where *τ*_*y*_ is the nominal yield shear stress of MR fluid, *R* is the radius of the sample, *S* is a plasticity number that is defined as *S* = *η*_*k*_*vR/h*^*2*^*τ*_*y*_,*S* was found to be always smaller than 0.05^12^, *η*_*k*_ is the Bingham plastic viscosity, *v* is the compressive velocity.

Williams et al.^19^ acquired the solution in two limit conditions: ① at *k → *0, namely MR fluids are Bingham fluids, and the compressive stress after some algebra can be written as

6 $$ \sigma = \frac{2R}{3}\frac{{\tau_{y} }}{{\left( {1 - \varepsilon } \right)^{2} }} $$

where *ε* is the compressive strain. Equation (6) can be transformed as

7 $$ \tau_{y} = \frac{2}{3}R\sigma \left( {1 - \varepsilon } \right)^{2} $$

The compressive stress of the MR fluid can be looked as driven by the field induced yield stress during compression. So the nominal yield shear stress *τ*_*y*_ can be calculated by the Eq. (7). ② at *k* = 1, namely MR fluids are Newtonian fluids, and the compressive stress can be written as *σ* = 3 *hη*_*k*_*r*_*0*_^2^*/2h*^3^*.* It is a pure squeeze flow of Newtonian fluid, and therefore does not belong to the scope of this study.

## Experimental setup

**Figure 2:**
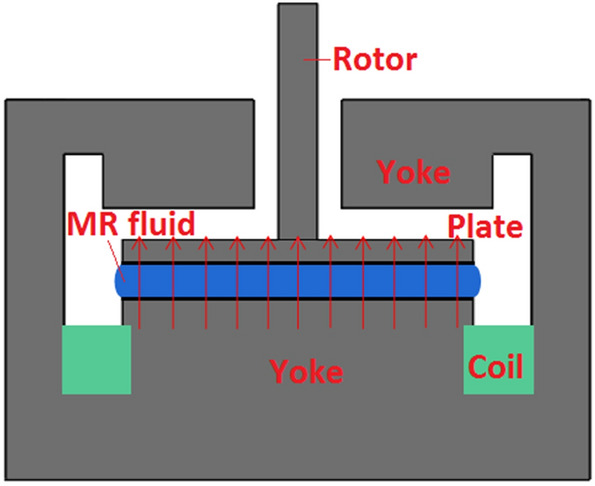
Configuration of the test system of the compressive properties of MR fluids under compression.

The MR fluid of MRF-2035, purchased from Ningbo Shangong Co. Ltd, China, was employed in this study. It is based on dimethyl silicon oil and iron powder with a particle volume fraction of about 35%. A MCR 302 commercial rheometer (Anton Paar, Graz, Austria) having a diameter of 20 mm for both plates was employed to investigate the squeeze flow behavior of MR fluid. The schematic diagram of MCR 302 rheometer is shown as in Fig. 2. The original gap distance *h*_*0*_ between the plates was set to 1.4, 1.2, 1.0, and 0.8 mm, respectively. At first, a certain amount of samples are placed between the parallel plates using a syringe. Then, after a current has been applied for 30 s, compression is carried out. The upper plate moved slowly down toward the static bottom plate under different compressive velocity *v* of 100, 75, 50, 25, and 10 μm/s, respectively. During the compression, the applied current is kept constant. At last, the applied current is turned off after the compression. It should be demagnetized after each experiment. The sample of MR fluid is injected again after each experiment.

**Figure 3:**
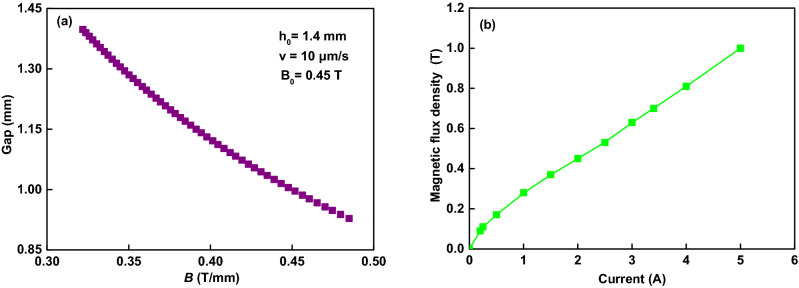
(**a**) The instantaneous magnetic flux density versus the gap; (**b**) The magnetic flux density changed with the applied current.

Corresponding to different *h*_*0*_, the instantaneous magnetic flux densities *B* can be kept to be 0.45 T/mm when the different magnetic flux density *B*_*0*_ of 0.37, 0.45, 0.53, and 0.63 T are applied, respectively. The compressive stress can be calculated as σ = 2*F*/π*R*^2^, where *R* is the radius of the plate. The compressive strain is defined as *ε* = (*h*_*0*_ − *h*)/*h*_*0*_, where *h* is the instantaneous distance between the two plates. The instantaneous magnetic flux density *B* during the compression process is calculated by *B* = *B*_*0*_*/h.* The instantaneous magnetic flux density *B* increases with the decreasing gap *h* during compression, as shown in Fig. 3a. The relationship between the applied current and the magnetic flux density is shown in Fig. 3b. The range of applied current generated by the coils is 0–5 A, and the magnitude of the magnetic field generates by the coils. The measuring range of force sensor is ± 50 N. All compression experiments reported here were run at constant slow velocity.The maximum of plasticity numbers *S* is 0.00024 ≪ 0.5. Reynolds numbers *Re* is 0.00126 ≪ 1. Therefore, the theoretical with lubrication theory and creeping flow is suitable for the current research. All experiments were done at room temperature, 23 °C.

## Results

### The compressive stress

**Figure 4:**
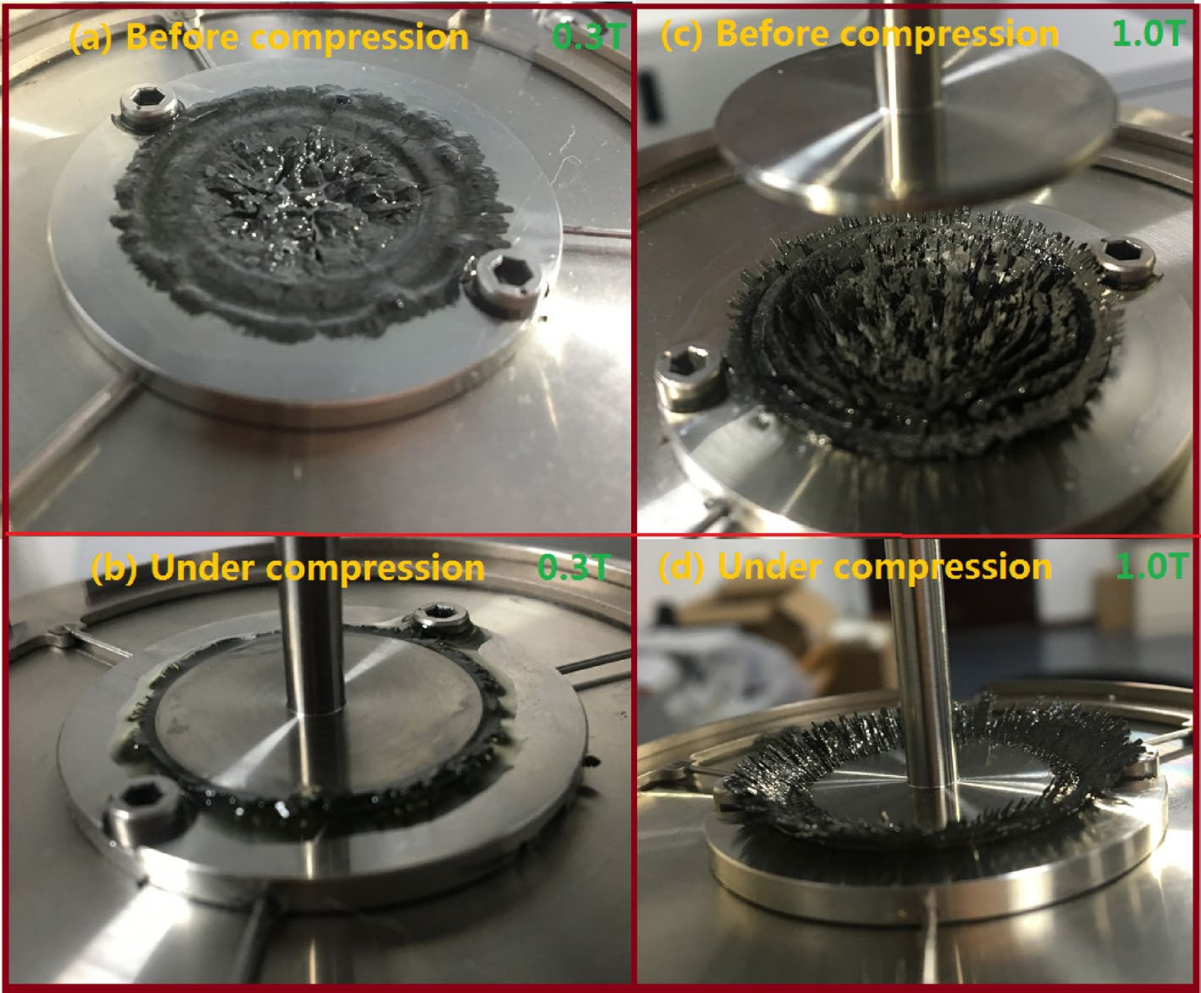
Photos of the MR fluids in the experiment. (**a**) Before compression with *B*_*0*_ = 0.3 T; (**b**) under compression with *B*_*0*_ = 0.3 T and *ε* = 0.7; (**c**) before compression with *B*_*0*_ = 1.0 T; (**d**) under compression with *B*_*0*_ = 1.0 T and *ε* = 0.7.

**Figure 5:**
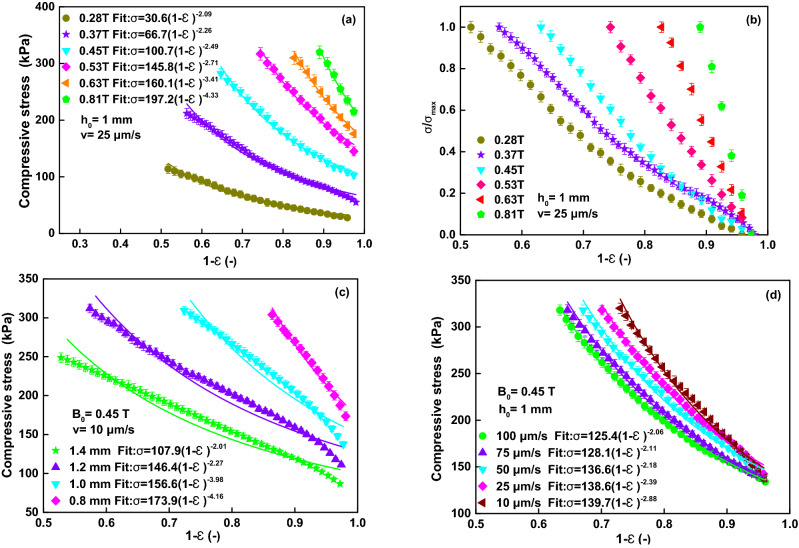
Compressive stress versus (1 − *ε*). Symbols: experimental data. Lines: fitting curve. (**a**) at *h*_*0*_ =1.0 mm *v* = 25 μm/s under different magnetic fields. (**b**) normalized compressive stress versus (1 − *ε*) under different magnetic fields. (**c**) at *B*_0_ = 0.45 T *v* = 10 μm/s under different original gap distances. (**d**) at *B*_0_ = 0.45 T and *h*_*0*_ =1.0 mm under different compressive velocity.

Figure 4 shows the photos of the MR fluid under compression. A series of unidirectional compression tests has been carried out with different magnetic field strengths, different original gap distances and different compressive velocities, as shown in Fig. 5. Figure 5a shows the compressive stress versus (1 − *ε*)/gap distance under different magnetic fields at *h*_*0*_ =1.0 mm and *v* = 25 μm/s. The different symbols represent the measured values and the solid lines represent the fitted values in Fig. 5a. The compressive stress increases quickly with the increase of the compressive strain or the decrease of the gap distance. Also, under a certain (1 − ε), the compressive stress at a higher applied magnetic field is obviously higher than that at a lower applied magnetic field. To compare the compressive characteristic under the different magnetic fields, the compressive stress can be normalized as σ/σ_max_, where σ is the instantaneous compressive stress and σ_max_ is the stable value, as shown in Fig. 5b. It shows that the compressive stress curves under different magnetic fields don’t overlapped each other. It means that the compressive stress was dependent of the applied current. Fitting the curves (the solid lines) with exponential functions, the indices are − 2.09, − 2.26, − 2.49, − 2.71, − 3.41, and − 4.33 for 0.28, 0.37, 0.45, 0.53, 0.63, and 0.81 T, respectively, as shown in Fig. 5a. *m* should be 2.5 according to Eq. (7) based on the squeeze flow theory. The change of *ε* determines the change of σ and *τ*_*y*_ during the compressive process according to Eq. (7). Many experimental reports about the relationship between the compressive stress σ and (1 − *ε*) show the exponent *m* about 2–5^12^.

The effect of different original gap distances (*h*_*0*_ =1.4, 1.2, 1.0, 0.8 mm) on the compressive stress of the MR fluid is studied at *B*_0_ = 0.45 T and *v* = 25 μm/s, as shown in Fig. 5c. The compressive stress at a smaller *h0* is obviously higher than that at a larger *h0* under the same compressive strain. Moreover, the exponent *m* increases with the decrease in the original gap distance. The exponential function for the curve (the solid lines) of *h*_*0*_ = 0.8 mm is σ = 173.9(1 − *ε*)^−4.16^. σ depends much strongly on *h*_*0*_ than that of *h*_*0*_ = 1.4 mm given by σ = 107.9(1 − *ε*)^−2.01^. Similar phenomena has been founded in previous research^14^.

The effect of different compressive velocity (*v* = 100, 75, 50, 25, 10 μm/s) on the compressive stress of the MR fluid is also studied at *B*_0_ = 0.45 T and *h*_*0*_ =1.0 mm, as shown in Fig. 5d. The compressive stress increases with the decrease of the compressive velocity under a constant compressive strain. The slower speed actually produces larger compressive stress at the same original gap. The exponent *m* is in range of 2.06–2.88, which almost agrees with the theoretical analysis. This result is in agree with that for MR fluids^14^ and is similar to that for ER fluids^21^. But comparing with the effect of the magnetic field or original gap distance, the compressive velocity has not strong influence on the changing of compressive stress during the compression.

In addition, Fig. 5a,c,d also show that the parameter *Kτ*_*y*_ increases with the increase the magnetic field strength, original gap distance and compressive velocity. According to Eq. (7), *K (2R/3)* is a constant. It suggests that the nominal yield shear stress *τ*_*y*_ actually increases with the increase of magnetic field strength, the decrease of original gap distance and compressive velocity. So, *τ*_*y*_ is a function of these quantities.

### The nominal yield shear stress

**Figure 6:**
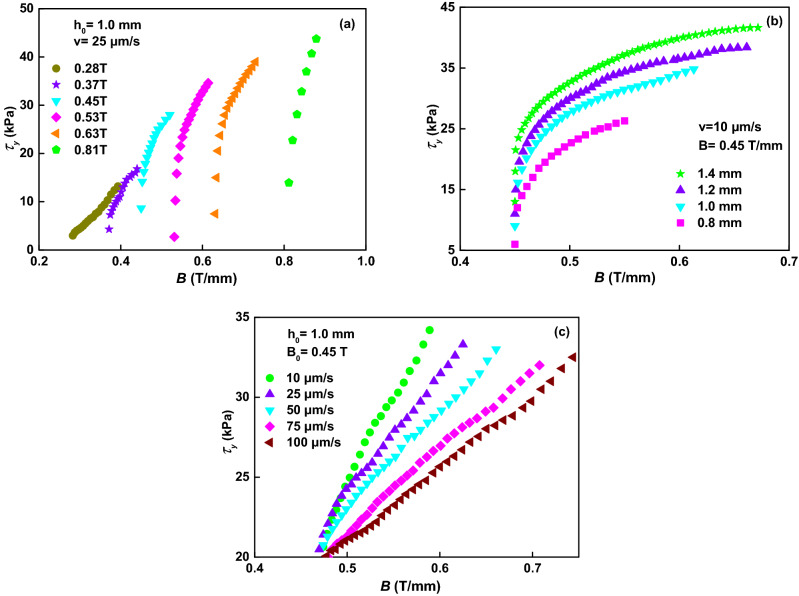
Nominal yield shear stress versus *B*. (**a**) at *h*_*0*_ =1.0 mm *v* = 25 μm/s under different magnetic fields. (**b**) at *B*_0_ = 0.45 T *v* = 10 μm/s under different original gap distances. (**c**) at *B*_0_ = 0.45 T and *h*_*0*_ =1.0 mm under different compressive velocity.

The yield shear stress of MR fluids is often described as *τ*_*d*_ = *αB*^*n*^, and *n* = 2 and *n* = 1.5 are in small and moderate magnetic fields, respectively^14^. The nominal yield shear stresses calculated according to Eq. (7) under different magnetic field strengths, different original gap distances and different compressive velocities are shown in Fig. 6. Figure 6a shows that curves are obviously different from each other. Fitting the curves (the solid lines) with exponential functions, the equations for the curves are *τ*_*d*_ = 610*B*^3.08^ (0.28 T), *τ*_*d*_ = 1107*B*^4.02^ (0.37 T), *τ*_*d*_ = 674*B*^4.72^ (0.45 T), *τ*_*d*_ = 720*B*^5.03^ (0.53 T), *τ*_*d*_ = 432*B*^5.91^ (0.63 T), and *τ*_*d*_ = 184*B*^10.61^ (0.81 T), respectively, which indicates that the exponents increase with the increase of the magnetic field strengths. The nominal yield shear stress is no longer proportional to 1.5 or 2 of the magnetic field strengths, but higher than the square. The increase in nominal yield shear stresses versus the instantaneous magnetic flux density gradually is accelerated. In fact, when the applied magnetic field is 0.81 T, the nominal yield shear stress increases at an almost straight line with the increase of the instantaneous magnetic flux density during the compression.

For different original gap distances (*h*_*0*_ = 1.4, 1.2, 1.0, and 0.8 mm), the nominal yield shear stress is shown in Fig. 6b when *v* = 10 μm/s and *B* = 0.45 T/mm. It also shows a trend that the exponent increases with the increase of the original gap distance. Ignoring the original instantaneous magnetic field, the curves are fitted with exponential functions (the solid lines). The exponents increase from 1.55 to 1.99 when the original gap distances increase from 0.8 to 1.4 mm, respectively. The nominal yield shear stress at a larger *h*_*0*_ is obviously higher than that at a smaller *h*_*0*_ under the same instantaneous magnetic field. In order to obtain the same original instantaneous magnetic field of 0.45 T/mm, the magnetic field should be applied for 0.63, 0.54, 0.45, and 0.36 T when the original gap distances is 1.4, 1.2, 1.0, and 0.8 mm, respectively. The nominal yield shear stress applied at a higher applied magnetic field is obviously higher but experienced a larger compressive strain than that applied at a lower applied magnetic field under a same final magnetic field. This result is contrary to the results for ER fluids by Tian et al*.*^22^, which showed that a smaller *h*_*0*_ can generate larger nominal yield shear stress at the same instantaneous magnetic field.

Figure 6c shows the nominal yield shear stress versus the instantaneous magnetic field of compressions under different compressive velocities at *B*_0_ = 0.45 T and *h*_*0*_ =1.0 mm. The exponents are 1.1 (*v* = 100 μm/s), 1.2 (*v* = 75 μm/s), 1.3 (*v* = 50 μm/s), 1.5 (*v* = 25 μm/s), and 2.2 (*v* = 10 μm/s), respectively, which means that the exponent increases with the decrease of the compressive velocity. The nominal yield shear stress depends strongly on the compressive velocity. A smaller compressive velocity result in a higher nominal yield shear stress at the same instantaneous magnetic field, showing that the slow compression can improve the nominal yield shear stress. This result is similar to the investigation for ER fluids by Tian et al.^21,23^.

### Comparison between experiments and calculations

**Figure 7:**
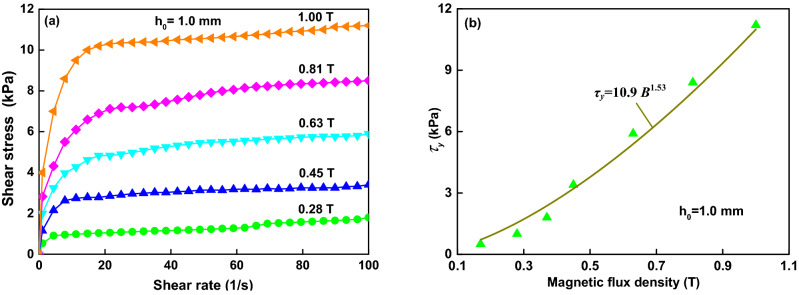
Results for the shear stress of an MR fluid at *h*_*0*_ =1.0 mm. (**a**) The shear stress curves of the MR fluid on different applied currents in a range of 0.28–1.0 T. (**b**) The fitted results for the yield shear stress of the curves under different magnetic fields.

**Figure 8:**
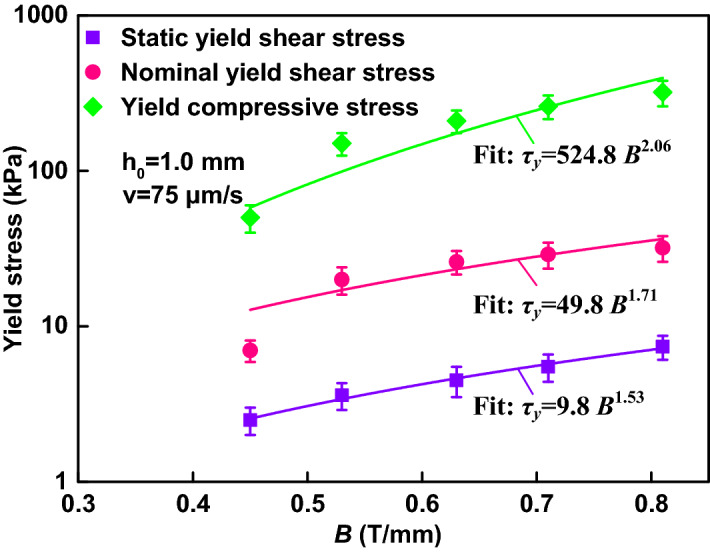
Comparison among yield compressive stresses, nominal yield shear stresses and static yield shear stresses at *h*_*0*_ =1.0 mm *v* = 75 μm/s.

In order to obtain the static yield shear stress (measured) of the same MR fluid, the shear stress versus shear rate at *h*_*0*_ = 1.0 mm and *B*_0_ = 0.45 T was measured by the same rheometer, as shown in Fig. 7a. The shear rate is between 0 and 100 s^−1^. Shearing the MR fluid with a gap distance of 1.0 mm, a yield shear stress of 10.8 kPa at 1.00 T has been obtained. Also, Fig. 7b shows that the static yield shear stress obtained for the MR fluid is proportional to the magnetic flux intensity *B* with an exponent of 1.53 (the fitting solid line), which agrees with the results of many former studies^3,5^. Figure 8 shows a comparative study on the yield stresses under shear and compression, including the static yield shear stress measured and the nominal yield shear stresses calculated according to Eq. (7). The yield compressive stress increases with the instantaneous magnetic flux intensity following a power law function with exponent 2.06, following a square relationship described by the dipolar models^6,24^. The nominal yield shear stresses is proportional to the instantaneous magnetic flux intensity with an exponent of 1.71. The yield compressive stress is about ten times of the nominal yield shear stress and four to five times of the static yield shear stress. Similar experimental results can be found in the work reported by Vicente et al.^12^. They reported the compressive curves under the constant magnetic field density during the decrease of the gap; the yield compressive stress is larger than the yield shear stresses, and the dynamic (nominal) yield stress is larger than the static yield stress; the yield compressive stress, the dynamic yield shear stress and the static yield shear stress increase with the magnetic field strength following a power law function with the exponents of 1.89, 0.97 and 1.62, respectively. Table 1 compares the present compression performance of the MR fluid under the instantaneous magnetic field and that^12^ under the constant. Similar experimental results of the nominal yield shear stress can also be found in the report of ER fluids at the same initial gap of 1 mm and the electric field of 0.25 kV by Tian et al*.*^21^ the nominal yield shear stress increases with the instantaneous electric field with the exponent of 2.73.

**Table 1:** Comparison between the present with the previous works about power law exponent *n* for the yield stress according to *τ*_*d*_ = *αB*^*n*^*.*

	Present works	Vicente et al.^12^
Magnetic field	Instantaneous	Constant
Yield compressive stresses	2.06	1.89
Nominal yield shear stresses	1.71	0.97
Static yield shear stresses	1.53	1.62

## Discussion

**Figure 9:**
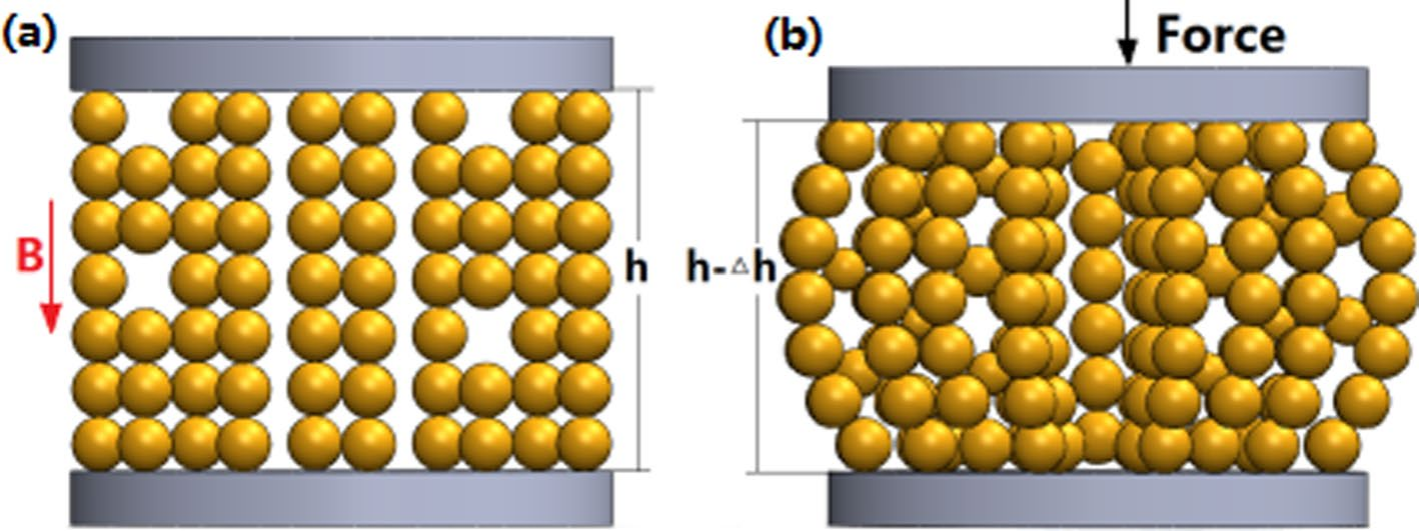
Schematic view of the formation of MR microstructure: (**a**) at the magnetic field; (**b**) at the magnetic field under compression.

Namely, similar to the sheared MR fluids involving the interaction forces between particles along the shear direction, the mechanical property of an MR fluid under compression is also greatly affected by its chain structure under magnetic fields. During the pre-compressions, the particles in the MR fluid form chains and columns when a magnetic field is applied to the MR fluid. The formation of MR microstructure at the magnetic field and under compression was modeled using a software package of Solidworks 18, as shown in Fig. 9a. According to the compression assisted-aggregation process of Tao et al*.*^25^*,* the weak points of MR chain at the chain’s ends will be repaired and the particle chains will aggregate easily into thick columns or more robust BCT lattice structure, as shown in Fig. 9b. Also, the gap between the two plates will be decreased during the compression, which induces the increase of magnetic field and the decrease of distance between the particles. The decreasing distance between the particles may result in the restructuring of particle chains into the more robust structure, which will bring greater resistance. The compressive stress and the nominal yield shear stress can be considered totally contributed by the resistances of the chains, while this resistance of the chains is determined by the applied magnetic field. Thus, the compressive stress and the nominal yield shear stress are determined by the applied magnetic field. The compressive results shown in Figs. 5a,c and 6a agree with this description.

The particle chains during the compression in MR fluids can be seen as compressing slim rods^14,22^. The rod strength *Fs* can be described as *Fs* = *J*(*d/L*)^2^, where *J* is a material parameter that is related to the particle interactions for MR fluids, *L* is the rod length and *d* is the rod diameter. So, a smaller chain length or the initial gap distance corresponds to a slightly higher rod strength or the compressive resistance when the diameter of the chains is constant. The relationship between the rod strength *Fs* or the compressive stress σ and the chain length *L* or the initial gap distance *h*_*0*_ shows an exponent of − 2, as shown in Fig. 5c at *h*_*0*_ = 1.4 mm. But the square relationship is not always satisfied, as shown in Fig. 5a,c,d. This is probably due to the fact that the assumption of single chain is not always applicable. Compression can generate the squeeze-strengthen effect reported by Zhang et al*.*^6^*.* Also, the rod diameter *d* will be increased when the particle chains aggregate from the chains into thick columns under compression, which also leads to a higher rod loadings or yield stress of MR chains and the increase of the material parameter *J.* Thus, the yield stress of MR fluids can be significantly strengthened under compression. Similar deviations have also been reported for MR fluids by Guo et al*.*^14^ and ER fluids by Tian et al*.*^22^.

During the slow compression, the compressive flow can be regarded as a two-phase flow of particles and carrier fluids. When shearing the MR fluid perpendicular to the magnetic field direction, there are old chains broken and new chains forming. Similarly, chains and columns may be destroyed and more robust structures will be formed during the compression, which is also a kind of squeeze-strengthen effect^6^. When the compression speed is high, the structure of the particle chain in MR fluids is damaged more seriously, resulting in the smaller compressive stress. The particles have enough time to form the more robust structures in the low compressive speed, which significantly enhanced the interconnectivity of chain structures. Deborah number is the ratio of relaxation time and testing time. The larger the Deborah number, the material properties are mainly close to solid. The relaxation time should have been increased faster than the increase of testing time because of the decrease of compressive velocity, as predicted by Tian et al*.*^21^. So it finally led to a higher Deborah number at a lower compressive speed and a more solid-like property of the MR fluid^21^. Similar experimental results can also be found in the work reported by Guo et al*.*^14^ at the particle concentration of the MR fluid of 30%. They also found that the squeeze velocity has a very small effect on the force-gap relationship at the particle concentration of the MR fluid of 15%. They explained that there must be a speed where the deformation of the particle structures proceeds at a rate too fast for stronger particle structures to be able to be reform during compression. The result is similar to the effect of the low compressive velocity in our analysis. Also, while shearing a MR fluid between two parallel plates, the volume of the MR fluid between plates is kept constant. During the compression, the volume fraction of particles increases as the liquid is being expelled. The magnetic permeability is proportional to the volume fraction of the magnetic particles in the fluid^26, 27^. So the greater the volume fraction of particles, more the magnetic particles will be magnetized to arrange themselves along the lines of magnetic flux. In consequence, there will be an increase in the fluid’s resistance to flow to some extent, i.e. the sealing effect, as shown in Fig. 4b,d. This sealing effect is similar to that reported by Mazlan et al.^10^. They found that the volume fraction of the particles will increase from 59.48 to 66.09% when the gap decreases from the initial gap size of 2 mm to 1.8 mm. The greater the volume fraction, the more the particles will be magnetized. They also found that the MR fluid’s viscosity will increase 1.561 times when the volume fraction of particles increases 6.61%. So there will be an increase in the fluid’s resistance to flow. It suggest that the much higher compressive resistance will be required to the movement of the carrier fluid.

The slope of yield shear stresses with the magnetic field theoretically predicted by continuous media theories is 1.5 or 2.0. The nominal yield stress calculated according to Eq. (7) varying with the magnetic field is higher than the theoretical value under different magnetic field. The nominal yield stresses are thought mainly contributed by the field induced resistance of the MR fluid. The deviation of nominal yield stresses from the description by Eq. (7) is dealt with the field induced yield stresses. Similar deviations have also been reported by Ruiz-López et al.^28^. They proposed a novel micromechanical model and the model can explains experimental findings for a wide range of concentrations and deformations where the classical continuum media theory tends to be not valid. Tian et al. found that the magnetic field dependency of nominal yield stresses in the squeez-flow subsequently demonstrates to be higher than the well known H^2.0^ predicted by continuous media theories. They also found that the description of compressive behavior of ER fluids with the continuous media theory and the Bingham model might not have reflected the essential attribute of the ER effect during compression^22^.

Furthermore, a pre-applied compression can effectively increase the yield shear stress of MR fluids. As shown above, the nominal yield shear stress is four to five times of the static yield shear stress under compression. Except for the strength of particle interaction, friction between the magnetizable particles happens and frictional forces between particles should be considered when the MR fluids are compressed^6^. So the yield shear stress of MR fluids can be determined by a semiempirical model, which includes the friction effect and the a modified magnetic dipole model^6^. This semiempirical model could qualitatively explain the increase in field-induced contact compressive stress and the nominal yield shear stress, and predict the squeeze-strengthen effect.

## Conclusion

In this investigation, compressions of magnetorheological (MR) fluids have been carried out under instantaneous magnetic fields at a slow compressive speed. Based on Bi-visous model, the yield strength of MR fluid was modeled by assuming that it was a transformed shear flow. With increasing applied magnetic field, the compressive stress increases, whereas with increasing initial gap distance and compressive velocity the compressive stress decreases. The nominal yield shear stresses of the compressed MR fluid under different influential factors have been calculated. The results shows that the nominal yield shear stresses increase in a power law as the applied magnetic field and the initial gap distance increase, the compressive velocity decreases. The yield compressive stress is about ten times of the nominal yield shear stress and four to five times of the static yield shear stress. The achieved results of the nominal yield shear stress with magnetic field seem to deviate from the prediction of dipole model. An explanation based on the chain structure aggregation effect, whose strength is affected by the field strength, the diameter and the length of the particles chains, the sealing effect and the friction effect by compression are considered. A unified model describing the compressive process of MR fluid under different influential factors still needs to be constructed.

## References

[1] Zhanga Y, Lib D, Cuib H, Yang J (2020). A new modified model for the rheological properties of magnetorheological fluids based on different magnetic field. J. Magn. Magn. Mater..

[2] Zhu W, Dong X, Huang H (2019). Iron nanoparticles-based magnetorheological fluids: A balance between MR effect and sedimentation stability. J. Magn. Magn. Mater..

[3] Kwon SH, Na SM, Flatau AB (2020). Fe-Ga alloy based magnetorheological fluid and its viscoelastic characteristics. J. Ind. Eng. Chem..

[4] Rahim MSA, Ismail I (2015). Review of magnetorheological fluids and nanofluids thermal behaviour. Mater. Sci. Eng..

[5] Tang X, Zhang X, Tao R, Rong Y (2000). Structure-enhanced yield stress of magnetorheological fluids. J. Appl. Phys..

[6] Zhang XZ, Gong XL, Zhang PQ (2004). Study on the mechanism of the squeeze-strengthen effect in magnetorheological fluids. J. Appl. Phys..

[7] See H (2003). Field dependence of the response of a magnetorheological suspension under steady shear flow and squeezing flow. Rheol. Acta.

[8] See H, Mackenzie S, Chua BT (2006). Effect of compression on the response of a magneto-rheological suspension. Korea-Aust. Rheol. J..

[9] Kulkarni P, Ciocanel C, Vieira SL (2003). Study of the behavior of MR fluids in squeeze, torsional and valve modes. J. Intell. Mater. Syst. Struct..

[10] Mazlan SA, Ekreem NB, Olabi AG (2007). “The performance of magnetorheological fluid in squeeze mode. Smart Mater. Struct..

[11] Mazlan SA, Ekreem KH, Olabi AG (2008). An investigation of the behaviour of magnetorheological fluids in compression mode. J. Mater. Process. Technol..

[12] Vicente JD, Ruiz-Lopez JA, Andablo-Reyes E (2011). Squeeze flow magnetorheology. J. Rheol..

[13] Ruiz-López JA, Hidalgo-Alvarez R, de Vicente J (2012). On the validity of continuous media theory for plastic materials in magnetorheological fluids under slow compression. Rheol. Acta.

[14] Guo C, Gong X, Xuan S (2013). Squeeze behavior of magnetorheological fluids under constant volume and uniform magnetic field. Smart Mater. Struct..

[15] Wang H, Bi C, Kan J (2011). The mechanical property of magnetorheological fluid under compression, elongation, and shearing. J. Intell. Mater. Syst. Struct..

[16] Liu ZY, Li F, Li XW (2021). Characteristic analysis and squeezing force mathematical model for magnetorheological fluid in squeeze mode. J. Magn. Magn. Mater..

[17] El Wahed AK, Sproston JL, Schleyer GK (2002). Electrorheological and magnetorheological fluids in blast resistant design applications. Mater. Des..

[18] John S, Chaudhuri A, Wereley NM (2008). A magnetorheological actuation system: Test and model. Smart Mater. Struct..

[19] Williams EW, Rigby SG, Sproston JL (1993). Electrorheological fluids applied to an automotive engine mount. J. Nonnewton. Fluid Mech..

[20] Covey GH, Stanmore BR (1981). Use of the parallel-plate plastometer for the characterization of viscous fluids with a yield stress. J. Non-Newton. Fluid Mech..

[21] Tian Y, Zhang M, Zhu X (2010). Ultrahigh yield stress in a general electrorheological fluid under compression. Smart Mater. Struct..

[22] Tian Y, Wen S, Meng Y (2003). Compressions of electrorheological fluids under different initial gap distances. Phys. Rev. E.

[23] Tian Y, Zhu X, Jiang J (2010). Structure factor of electrorheological fluids in compressive flow. Smart Mater. Struct..

[24] Bossis, G., Volkova, O., Lacis, S. & Meunier A. Magnetorheology: fluids, structures and rheology. In *Ferrofluids. Lecture Notes in Physics*, Vol. 594 (ed. Odenbach, S.) (Springer, 2002).

[25] Tao R (2001). Super-strong magnetorheological fluids. J. Phys. Condens. Matter.

[26] Noresson V, Ohlson NG (2001). A critical study of the Bingham model in squeeze-flow mode. Mater. Des..

[27] Simon TM, Reitich F, Jolly MR, Ito K, Banks HT (2001). The effective magnetic properties of magnetorheological fluids. Math. Comp. Model..

[28] Ruiz-López AJ, Hidalgo-Alvarez R, Vicente J (2016). A micromechanical model for magnetorheological fluids under slow compression. Rheol. Acta.

